# Akirin2 promotes slow myosin heavy chain expression by CaN/NFATc1 signaling in porcine skeletal muscle satellite cells

**DOI:** 10.18632/oncotarget.15374

**Published:** 2017-02-16

**Authors:** Xiaoling Chen, Yanliu Luo, Zhiqing Huang, Guangmang Liu, Hua Zhao

**Affiliations:** ^1^ Key Laboratory for Animal Disease-Resistance Nutrition of China Ministry of Education, Institute of Animal Nutrition, Sichuan Agricultural University, Chengdu, Sichuan 611130, P. R. China

**Keywords:** Akirin2, slow myosin heavy chain, CaN/NFATc1, porcine skeletal muscle satellite cells

## Abstract

The objective of this study was to evaluate the effect of Akirin2 on slow myosin heavy chain (slow MyHC, MyHC I) gene expression and its molecular mechanisms. In this study, we showed that the protein expression of Akirin2 in pig slow oxidative *Psoas major* muscle is higher than that in fast glycolytic *tibialis anterior* muscle, suggesting that Akirin2 may play a role in myofiber typing. Knockdown of Akirin2 decreased the MyHC I expression and the calcineurin (CaN) activity, and also decreased the expressions of NFATc1 and MCIP1.4. Conversely, overexpression of Akirin2 got the opposite results. Furthermore, inhibition of CaN or knockdown of NFATc1 attenuated Akirin2 overexpression-induced upregulation of MyHC I. Together, these results demonstrate that Akirin2 promotes MyHC I expression via CaN/NFATc1 signaling pathway in porcine skeletal muscle satellite cells.

## INTRODUCTION

Skeletal muscle is a heterogeneous tissue composed of a large variety of fiber types classified according to their myosin heavy chain (MyHC) isoforms. There are four different types of muscle fibers: type I (expressing slow MyHC, namely MyHC I), type IIa (expressing MyHC IIa), type IIb (expressing MyHC IIb), and type IIx (expressing MyHC IIx). Type I fiber, also known as slow oxidative muscle fiber, is believed to be positively associated with meat quality [1, 2]. The intramuscular fat (IMF) content (marbling) is also associated with a higher content of oxidative fibers in skeletal muscle [3].

Akirin2 is a highly conserved nuclear protein [4]. Numerous reports have described the involvement of Akirin2 in the embryonic development and immune defense function [4–7]. However, the role of Akirin2 in skeletal muscle is far less understood. Currently, Akirin2 has been reported to be located within genomic region of a quantitative trait locus for marbling, and has been shown to be differentially expressed in *musculus longissimus* muscle of low-marbled and high-marbled steer groups [8]. A number of studies showed that Akirin2 is associated with marbling based on single nucleotide polymorphisms analysis and may be considered as a positional functional candidate for the gene responsible for marbling [9–12].

Little research has been conducted on the role of porcine Akirin2 (pAkirin2). The pAkirin2 gene was cloned and its tissue distribution in pig was examined [13]. We also proved that recombinant pAkirin2 significantly increased the proliferation of C2C12 myoblasts [14]. In C2C12 skeletal muscle cells, we found that Akirin2 could increase the mRNA expression of MyHC I and NFATc1 [15]. To further our knowledge of pAkirin2 in skeletal muscle, the aim of this study was to investigate whether Akirin2 modulates MyHC I expression via calcineurin/NFATc1 signaling pathway in porcine skeletal muscle satellite cells.

## RESULTS

### Endogenous Akirin2 gene expression in skeletal muscle

**Figure 1:**
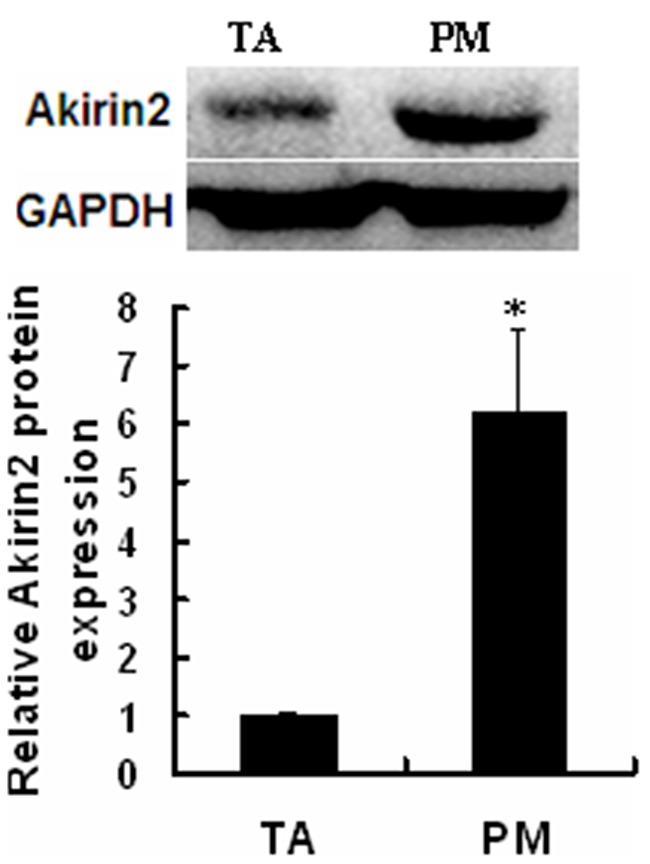
Expression of endogenous Akirin2 gene Western blot was used to analyze the Akirin2 protein expression in pig slow oxidative Psoas major (PM) muscle and fast glycolytic tibialis anterior (TA) muscle. Data were presented as means ± SE (n=3). *P < 0.05.

The endogenous expression of Akirin2 was analyzed in pig skeletal muscle. Western blot analysis with an anti-Akirin2 antibody prepared in our lab showed that Akirin2 protein level in the pig slow oxidative *Psoas major* muscle (PM) was about six-fold higher than that in the fast glycolytic *tibialis anterior* muscle (TA) (Figure 1), suggesting that Akirin2 may play a role in muscle fiber typing.

### Akirin2 promotes MyHC I expression in porcine skeletal muscle satellite cells

**Figure 2:**
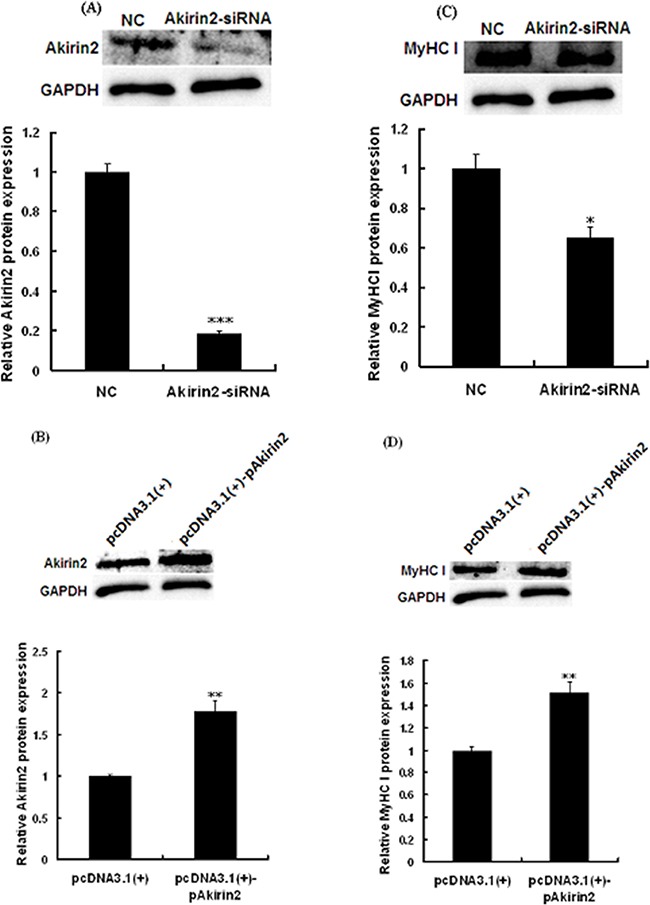
Akirin2 regulates the expression of MyHC I in porcine skeletal muscle satellite cells Porcine skeletal muscle satellite cells were transfected with negative control siRNA (NC), Akirin2 siRNA, pcDNA3.1(+) or pcDNA3.1(+)-pAkirin2 when cells reached about 80% confluence and induced to differentiate for 6 days before analysis. Western blot analysis was used to analyzed the Akirin2 **A-B**. and MyHC I **C-D**. protein expression in porcine skeletal muscle satellite cells. Data were presented as means ± SE (n=3). *P < 0.05 and **P < 0.01 as compared with control.

To test whether Akirin2 affects MyHC I expression in porcine skeletal muscle satellite cells, RNA interference and gene overexpression technologies were employed. Western blot analysis showed that knockdown of Akirin2 by siRNA significantly repressed the protein expressions of Akirin2 (Figure 2A) and MyHC I (Figure 2C). However, Akirin2 overexpression presented an increase in Akirin2 (Figure 2B) and MyHC I protein expressions (Figure 2D), suggesting that Akirin2 promotes MyHC I expression.

### Effect of Akirin2 on the protein expression of NFATc1 and CaN activity in porcine skeletal muscle satellite cells

**Figure 3:**
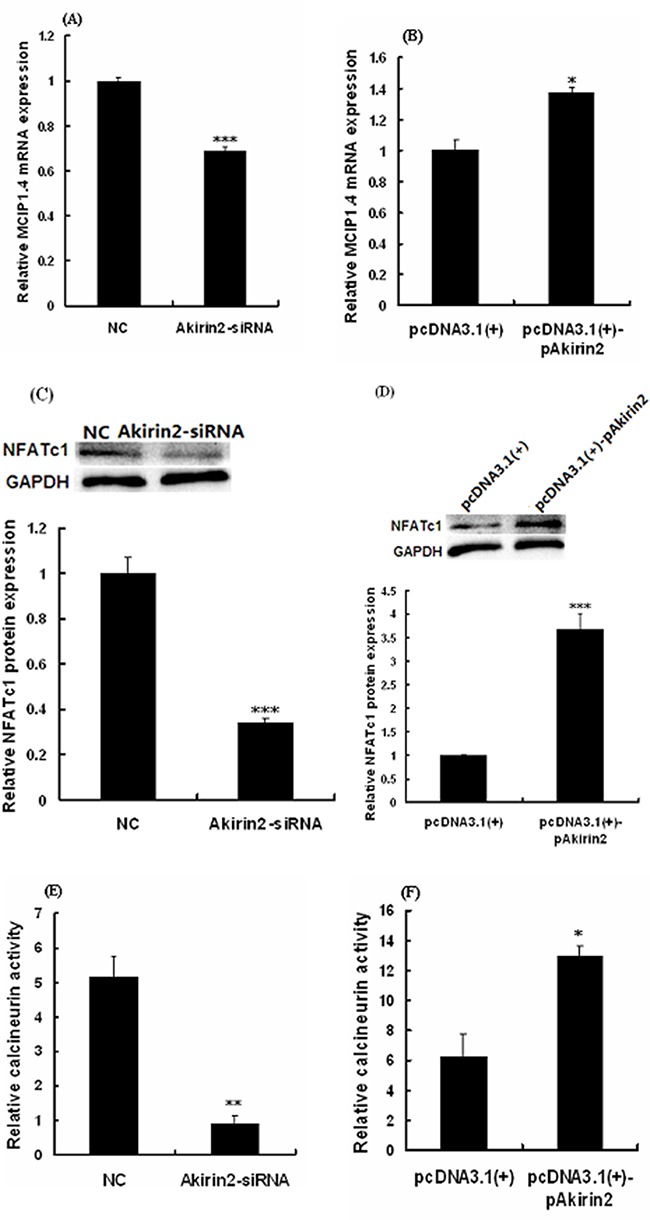
Effects of Akirin2 on the NFATc1 expression and CaN activity Porcine skeletal muscle satellite cells were cultured, transfected and induced to differentiate as described in Figure 2. Real-time quantitative PCR was used to analyze the MCIP1.4 **A-B**. mRNA expression in porcine skeletal muscle satellite cells. Western blot was used to analyze to the protein expression of NFATc1 **C-D**. in porcine skeletal muscle satellite cells. Calcineurin activity was analyzed by Calcineurin Cellular Activity Assay Kit **E-F**. Data were presented as means ± SE (n=3). *P < 0.05 and ***P < 0.001 as compared with control.

As shown in Figure 3A and 3B, silencing of Akirin2 by siRNA and overexpression of Akirin2 significantly decreased and increased modulatory calcineurin interacting protein exon 4 isoform (MCIP1.4, a NFATc1 target) protein expression in porcine skeletal muscle satellite cells, respectively. Analysis also indicated that Akirin2 affected NFATc1 protein expression (Figure 3C-3D), suggesting that NFATc1 is downstream of Akirin2.

To further determine the relationship between Akirin2 expression and CaN activity, we examined the effects of Akirin2 silencing and overexpression on the CaN activity. The results showed that silencing of Akirin2 significantly decreased the CaN activity (Figure 3E), while overexpression of Akirin2 got the opposite result (Figure 3F).

### Akirin2 promotes MyHC I expression via CaN/NFATc1 signaling

**Figure 4:**
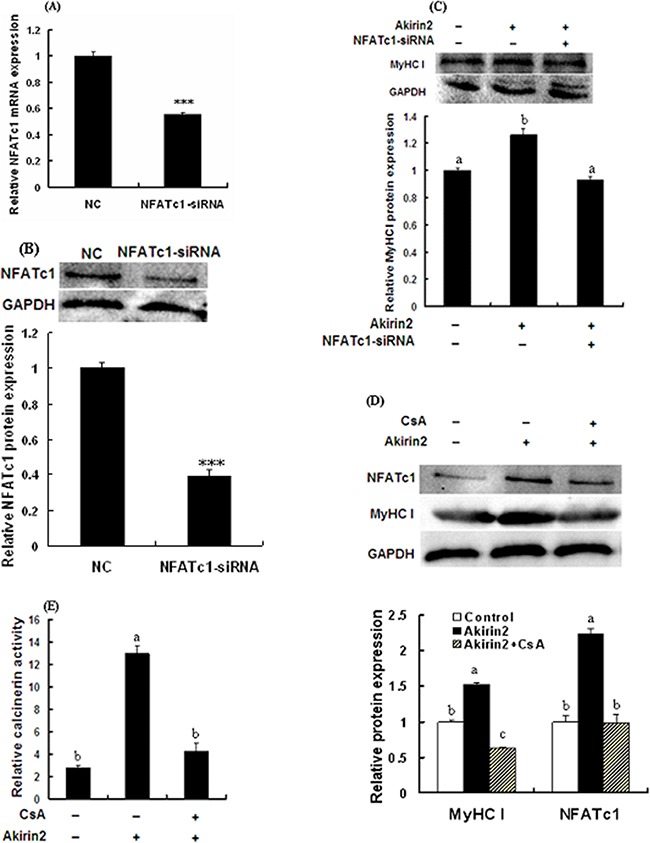
Akirin2 regulates the expression of MyHC I via CaN/NFATc1 signaling in porcine skeletal muscle satellite cells Cells were cultured, transfected and induced to differentiate as described in Figure 2. Real-time quantitative PCR was used to analyze the NFATc1 mRNA expression in porcine skeletal muscle satellite cells. Western blot was used to analyze to the protein expression of NFATc1 and MyHC I in porcine skeletal muscle satellite cells. Real-time quantitative PCR analysis indicated that the siRNA induced a 45% downregulation of NFATc1 mRNA expression **A**. Western blot analysis indicated that the protein expression level of NFATc1 was also significantly decreased **B**. Silencing of NFATc1 by siRNA inhibited Akirin2 overexpression-induced upregulation of MyHC I **C**. Inhibition of CaN by CsA abolished Akirin2 overexpression-induced upregulation of MyHC I and NFATc1 protein expressions **D**. and increase in the CaN activity **E**. Data were presented as means ± SE (n=3). ***P < 0.001. Value with different letters indicates significantly difference (P < 0.05).

To test if NFATc1 was involved in Akirin2-induced promotion ofMyHC I expression, NFATc1 was knocked down by siRNA in porcine skeletal muscle satellite cells, and the expression of MyHC I was analyzed. As shown in Figure 4A and 4B, both the mRNA and protein expression levels of NFATc1 were significantly decreased. As shown in Figure 4C, knockdown of NFATc1 abolished Akirin2 overexpression-induced upregulation of MyHC I.

As shown in Figure 4D and 4E, CaN inhibitor CsA significantly attenuated Akirin2 overexpression-induced upregulation of NFATc1 and MyHC I expressions and increase in the CaN activity. Taken together, these results showed that Akirin2-mediated upreguation of MyHC I is dependent on CaN/NFATc1 signaling.

## DISCUSSION

Meat quality, one of the most important economic traits in farm animals, is controlled by multiple genes and affected by many factors. One of the main factors is the muscle fiber characteristics because skeletal muscles mainly consist of muscle fibers [16]. There is a lot of evidence to suggest that increasing the percentage of type I fibers is related to meat quality improvement [1, 2, 17]. Therefore, it is necessary to identify novel candidate genes for improving meat quality by regulating muscle type I fiber expression.

TA muscle is a typical fast muscle, while PM muscle is a typical slow muscle [18]. We found that higher levels of Akirin2 protein were detected in slow oxidative PM muscle compared to fast glycolytic TA muscle (Figure 1). In the present study, we first evaluated the effect of Akirin2 on MyHC I expression in porcine skeletal muscle satellite cells. The data showed that Akirin2 affected MyHC I in both gene silencing and overexpression of Akirin2 studies (Figure 2). Additional molecular mechanism experiments revealed that Akirin2 acted through CaN/NFATc1 signaling (Figure 3).

Previous studies have described calcineurin-NFAT signaling pathway regulating oxidative muscle fiber type transition in skeletal muscle [19–22]. Calcineurin is activated by calcium/calmodulin and promotes NFAT nuclear translocation where NFAT induces the slow-twitch phenotype in skeletal muscle [22, 23]. The NFAT transcription factor family consists of five members NFATc1, NFATc2, NFATc3, NFATc4 and NFATc5. NFATc1 through NFATc4 are activated by calcineurin and expressed in skeletal muscle [24]. NFATc1 may be control fiber type composition and plays an important role in fast-to-slow fiber type switching [19, 25]. In this study, we present the first evidence that Akirin2 mediated the MyHC I expression via NFATc1 signaling since knockdown of Akirin2 not only downregulated MyHC I, but also decreased the expressions of NFATc1 and its downstream target gene MCIP1.4, whereas overexpression of Akirin2 got the opposite results (Figure 3). The expression level of MCIP1.4 reflects the calcineurin activity and the promoter of MCIP1.4 includes a cluster of NFAT-binding sites, and hence MCIP1.4 appears a direct downstream target of the calcineurin/NFAT pathway [26–28]. Furthermore, an increase in MCIP1.4 mRNA level reflects the switch from fast to slow-type MyHC isoforms [28]. We therefore measured the level of MCIP1.4 mRNA and found overexpression of Akirin2 significantly increased MCIP1.4 mRNA level (Figure 3B), whereas knockdown of Akirin2 got the opposite result (Figure 3A). These results suggested that the MCIP1.4 mRNA level may be mirror the calcineurin activity and MCIP1.4 plays an important role in affecting slow type fiber gene expression. Furthermore, silencing of NFATc1 by siRNA inhibited the effect of Akirin2 overexpression on MyHC I expression (Figure 4C). CaN activity assay and Western blot analysis further indicated that Akirin2 promotes MyHC I expression via CaN/NFATc1 signaling pathway.

In conclusion, we reported that Akirin2 promotes MyHC I expression in porcine skeletal muscle satellite cells. This function is dependent on CaN/NFATc1 signaling. The present study shed new light on the role of Akirin2 in pigs.

## MATERIALS AND METHODS

### Reagents

The calcineurin inhibitor cyclosporin A (CsA) was obtained from Amresco (Solon, OH, USA) and resolved in DMSO, then used at 5 μM. NFATc1 antibody was obtained from Cell Signaling Technology (Danvers, MA, USA). GAPDH antibody was obtained from Santa Cruz Biotechnology (Santa Cruz, CA, USA). MyHC I antibody (BA-F8) was purchased from the Developmental Studies Hybridoma Bank (DSHB, Iowa City, IA, USA). Calcineurin Cellular Activity Assay Kit was purchased from Solarbio (Beijing, China).

### Animals and tissue sample collection

Three 10-week-old female Duroc × Landrace × Yorkshire (DLY) pigs were slaughtered at body weight of 31-31.6 kg in a humane manner according to protocols approved by the Animal Care Advisory Committee of Sichuan Agricultural University. The psoas major (PM) muscle and the tibialis anterior (TA) muscle were removed and immediately snap frozen in liquid nitrogen before being stored at −80 °C for protein extraction.

### Isolation of porcine skeletal muscle satellite cells and cell culture

Porcine skeletal muscle satellite cells were isolated from longissimus lumborum muscle of 3-day-old male DLY pigs. The pigs obtained from Sichuan Zhengyuan swine industry Co., Ltd. (Chengdu, Sichuan, China) were slaughtered according to protocols approved by the Animal Care Advisory Committee of Sichuan Agricultural University. Briefly, muscle was digested with 0.2% collagenase type II (Sigma, St. Louis, MO, USA) and then filtered successively through 200-mesh and 400-mesh cell sieves. The collected cells were purified by differential adhesion method. The resulting cells were cultured in Dulbecco's modified Eagle's medium (DMEM)/F12 (Invitrogen, Carlsbad, CA, USA) comtaining 20% fetal bovine serum (FBS), 100 U/mL penicillin and 100 μg/μL streptomycin at 37 °C in a humidified 5% CO_2_ atmosphere. The cells were identified by immunofluorescence staining with Pax7 antibody (DSHB).

### The siRNA and plasmid transfection

In this study, the siRNA sequence targeting NFATc1 was designed and synthesized by GenePharm (Shanghai, China). The sense strand of the NFATc1 siRNA was 5′-GGACCAGGAGUUCGACUUUTT-3′, and the antisense strand was 5′-AAAGUCGAACUC CUGGUCCTT-3′; The sense strand of the negative control siRNA was 5′-UUCUCCGAACGUGUCACGUTT-3′, and the antisense strand was 5′-ACGUGACACGUU CGGAGAATT-3′. Porcine skeletal muscle satellite cells were transfected with 50 nM siRNA for NFATc1, 50 nM negative control siRNA, 2 μg pcDNA3.1(+)-Akirin2 plasmid (Chen at al., 2012) or 2 μg empty pcDNA3.1(+) vector by using Lipofectamine 3000 (Invitrogen) according to the manufacturer's instructions.

### Cell treatment

To determine whether the Akirin2 affects the expression of MyHC I via the calcineurin/NFATc1 signaling pathway, we treated the porcine skeletal muscle satellite cells with 5 μM CsA or the siRNA for NFATc1, and analyzed the protein expressions of NFATc1 and MyHC I by Western blot.

### RNA extraction and real-time quantitative PCR

Total RNA was extracted from the cells using the RNAiso Plus reagent (TaKaRa, Dalian, China) according to the manufacture's instructions. One microgram of total RNA was transcribed into a single strand cDNA by using PrimeScript® RT reagent Kit with gDNA Eraser (TaKaRa). For real-time quantitative PCR, the SYBR select Master Mix (Applied Biosystems, Foster, CA, USA) was used in an ABI 7900HT Real-time PCR system. The gene specific primers used are as follows: porcine MCIP1.4 (forward) 5′-GGGCCAAATTTGAATCCC-3′ and (reverse) 5′-GGGTTGCTGAAGTTGATTCTG-3′; porcine GAPDH (forward) 5′-ACACTGAGGACCAGGTTGTG-3′ and (reverse) 5′-GACGAAGTGGTCGTTGAGGG-3′. The PCR cycling conditions used were: 45 cycles at 95 °C for 15 s and 60 °C for 30 s. Relative gene expression was calculated by the comparative Ct method [29]. GAPDH served as an internal standard.

### Western blot

Western blot analysis was performed as previously described by Chen et al. [30]. Briefly, equal amounts (30 μg) of protein of each sample were subjected to 10% sodium dodecyl sulfate-polyacrylamide gel electrophoresis. Following transfer of the proteins to a nitrocellulose membrane, the membrane was incubated in 3% non-fat milk in TBS-0.1% Tween-20 and then incubated overnight with primary antibody at 4 °C, followed by horseradish peroxidase-linked secondary antibodies (Santa Cruz Biotechnology). The proteins were visualized by Clarity^TM^ Western ECL Substrate (Bio-Rad, Hercules, CA, USA). GAPDH is considered as a housekeeping protein. The density of protein bands was measured using Gel-Pro Analyzer 4.2 software (Media Cybernetics, Rockville, MD, USA).

### Calcineurin activity analysis

Cells were harvested and rinsed three times using PBS. Cells were lysed in ice-cold lysis buffer (1% Triton X-100) for 30 min, and the lysates were used immediately for calcineurin (CaN) activity assay. CaN activity was measured with the Calcineurin Cellular Activity Assay Kit according to the manufacturer's instructions. Specific CaN activity was calculated by normalization to the total protein content. Data are expressed as U per mg protein.

### Statistical analysis

All data are presented as mean ± SE. The data were subjected to one-way ANOVA analysis and Tukey's tests using SPSS 11.0 software. Differences were accepted as significant at *P* < 0.05.
